# Association of SNP rs9939609 in *FTO gene* with metabolic syndrome in type 2 diabetic subjects, rectruited from a tertiary care unit of Karachi, Pakistan

**DOI:** 10.12669/pjms.311.6524

**Published:** 2015

**Authors:** Asher Fawwad, Iftikhar Ahmed Siddiqui, Nimra Fatima Zeeshan, Syed Muhammad Shahid, Abdul Basit

**Affiliations:** 1 Asher Fawwad, M.Phil. Assistant Professor, Senior Research Scientist, Research Department, Baqai Institute of Diabetology and Endocrinology, Baqai Medical University, Plot No. 1-2, II-B, Nazimabad No2, Karachi-74600, Pakistan.; 2 Iftikhar Ahmed Siddiqui, PhD. Chairman& Professor of Biochemistry, Department of Biochemistry, Baqai Medical University, Pakistan.; 3 Nimra Fatima Zeeshan, B.E (Bio Engineering). Research Officer, Research Department, Baqai Institute of Diabetology and Endocrinology, Baqai Medical University, Plot No. 1-2, II-B, Nazimabad No2, Karachi-74600, Pakistan.; 4 Syed Muhammad Shahid, Assistant Professor,The Karachi Institute of Biotechnology & Genetic Engineering (KIBGE), University of Karachi, Karachi-75270, Pakistan; 5 Abdul Basit, FRCP. Professor of Medicine, Department of Medicine, Baqai Institute of Diabetology and Endocrinology, Baqai Medical University, Plot No. 1-2, II-B, Nazimabad No2, Karachi-74600, Pakistan.

**Keywords:** Association study, FTO-rs9939609, type 2 diabetes, Metabolic Syndrome

## Abstract

**Objective:**

To determine the association of SNP in *FTO* gene, rs9939609, with Metabolic Syndrome (MS) in type 2 diabetic subjects at a tertiary care unit of Karachi, Pakistan.

**Methods:**

We genotyped *FTO *rs9939609 SNP in 296 patients with type 2 diabetes from the Out Patient Department (OPD) of Baqai Institute of Diabetology and Endocrinology (BIDE). MS was defined on the basis of International Diabetes Federation (IDF) and National Cholesterol Education program (NCEP) criterion. Association between the rs9939609 SNP and MS was tested through chi-square and Z-tests by using odds ratio (OR) with 95% confidence intervals.

**Results:**

The frequency of MS as defined by IDF criterion was significantly higher in female subjects as compared to male subjects (p= 0.006). Carriers of ≥ 1 copy of the rs9939609 A allele were significantly more likely to had MS (69.6%) than non-carriers (30.4%), corresponding to a carrier odds ratio (OR) of 0.52 (95% confidence interval [CI] (0.29-0.93), with a similar trend for the ATP III-defined MS.“A” allele carriers under dominant model, carry all the criterion of MS more significantly as compared to non-carriers.

**Conclusion:**

The FTO rs9939609 SNP was associated with an increased risk for Metabolic Syndrome in type 2 diabetic populations at a tertiary care unit of Karachi, Pakistan.

## INTRODUCTION

Metabolic Syndrome (MS), is defined as a constellation of potential risk factors like obesity, hypertension, hyperglycemia, dyslipidemia and insulin resistance. It is a global health problem and a substantial economic burden for both developing and developed countries.^1^ MS escalates the risk of development in patients with type 2 diabetes by five times and doubles the risk of cardiovascular diseases.^2^

MS currently affects about 25% of the adult population in Europe^2^and 10%-19% ofAsian population.^4^ With genetic predisposition, sedentary lifestyle, higher intake of total energy. Specific macronutrients may contribute to the development of MS. The data from Pakistan showed a prevalence rate of 18%-46% while 46%-75% Pakistani patients with diabetes were found to have MS.^4^^,^^5^ Although obesity and MS do not completely overlap, obesity is considered the core of MS.^3^

Each essential and defining component of MS has been previously found to be associated with genetic factors signifying that genetic basis might trigger and underlie the overall MS not only independently but also through a more complex interactions and cascades.^7^ Several genome-wide association studies (GWASs) identified obesity susceptibility loci.^7^A potential factor underlying genetic susceptibility to MS is the *FTO *(fat mass and obesity associated) gene^8^The human *FTO(OMIM no. 610966)* is located on chromosome 16q12.2 which consists of 9 exons and spans 410,507 bp.^9^ The rs9939609 single nucleotide polymorphism (SNP)with nucleotide position “53786615”,identified with the T to A missense mutation,^10^ located within the first intron of *FTO* was found to have a strong association with body mass index (BMI).^4^It is a common variant that is widely studied in different populations.^11^The AA homozygous genotype results in an average increase of ~3 kg in body weight or one BMI unit in the subjects compared to the TT genotype.^10^ This SNP is also found to be associated with central obesity and patients with type 2 diabetes.^12^ The reported results are not consistent in different ethnic population. In Europeans, *FTO *increased the risk of patients with type 2 diabetes through its impact on BMI while the reports from South Asian population showed that the risk of patients with type 2 diabetese associated with SNP of *FTO* gene was not dependent on BMI.^12^

Since obesity is one of the key features of MS, we wondered whether the association of SNP at *FTO*SNP could be found with MS. To best of our knowledge no study from Pakistan reported the association of MS with *FTO* gene in diabetic population. The aim of the study was to see the association of SNP in *FTO* gene, rs9939609, with metabolic syndrome at a tertiary care unit of Karachi, Pakistan.

## METHODS

This study was conducted in the Out Patient Department of Baqai Institute of Diabetology and Endocrinology (BIDE). The current study is a part of a case control study, intending to see the association of *FTO* gene with type 2 diabetes. The study duration was from March 2011 to May 2013. Karachi, the largest metropolitan city in Pakistan, is a representative of all major ethnic groups.^13^All patients with type 2 diabetes, who gave written informed consent during the study period were considered eligible to participate in the study. Patients with type 2 diabetes with severe cardiac, renal and hepatic diseases, patients with type 1 diabetes, pregnant females, older patients and hospitalized individuals were excluded. Ethical approval for the study was obtained from Institutional Review Board (IRB) of BIDE.

Measurements of waist circumference (WC), Body mass Index (BMI), blood pressure (BP), fasting analyses including venous plasma glucose, serum cholesterol, triglycerides (TG), low-density lipoprotein (LDL) cholesterol, High density lipoprotein cholesterol (HDL)and HbA1c were performed as per standard procedures. As per guidelines for the Asian population, BMI was categorized into normal weight between 18-22.9 kg/m^2^, overweight between 23-24.9 kg/m^2^and obese≥ 25 kg/m^2^.^14^

Genomic DNA was extracted out from whole blood using Phenol-Chloroform Method.^15^ Investigations for genotyping of rs9939609 polymorphism at FTO gene was performed by Amplification Refractory Mutation System (ARMS PCR).^16^ A fragment of DNA containing rs9939609 polymorphism was amplified using specific set of primers. A pair of outer and a pair of inner primers were designed using Primer 3 software (http://primer3.ut.ee/) for amplification of specific regions. Primers sets were

Fout:5’GTTCTACAGTTCCAGTCATTTTTGACAGC3’;

Rout:5’AGCCTCTCTACCATCTTATGTCCAAACA3’, 

Fin:5’TAGGTTCCTTGCGACTGCTGTGAATATA3’,

Rin: 5’GAGTAACAGAGACTATCCAAGTGCATCTCA3’.

 The ARMS PCR was carried out in a multi block system (Thermo Hybaid, USA). Total PCR reaction volume was 15 μl, containing,1xPCR amplification buffer, 100 ng genomic DNA, 0.6 mM/each primer, 1.5 mM MgCl2, 0.2 mM/each dNTPs and 2.5 U Taq DNA polymerase. Double stranded DNA was denatured at 94°C for 4 minutes, followed by 35 cycles of 94°C for 35 seconds. Annealing was done at primer-specific annealing temperature 65°C for 45 seconds and 72°C for 45 seconds and final extension at 72°C for 10 minutes. About 35 cycles were set for each PCR reaction. The size of outer common amplicon of rs 9939609 was 436bp whereas, the size of TT (homozygous allele protective allele), AA (homozygous risk allele) and AT (heterozygous allele) was 293 bp, 201bp and 293bp respectively. The amplified PCR products were observed on 2% agarose gel. A 5 µl aliquot of the PCR products was mixed with 2 µl of 6X gel loading buffer and run to horizontal 2% agarose gel electrophoresis assembly. The gel was stained with 10% ethidium bromide and visualized using gel documentation system (Bio Rad).

We used International Diabetes Federation (IDF)^17^ and National Cholesterol Education program (NCEP- ATP III)^18^ definition for the diagnostic criteria of metabolic syndrome (Table-I).

Statistical analyses were performed with the SPSS 13.0.Student t-test and ANOVAwere used to compare means of baseline variables while Chi-square and Z-tests were used to analyze the association of MS and its components with *FTO* genotypes by using odds ratio (OR) with 95% confidence intervals.

Hardy Weinberg Equilibrium test (HWE) was applied to determine the variation in distribution of alleles and genotypes within the concerned population. Allelic frequencies were calculated by gene counting .We used the dominant model of SNP rs9939609 in *FTO* gene (TT vs. AA/AT) for the analysis purpose

## RESULTS

A total of 296 type 2 diabetic subjects were recruited of which 179 were males and 117 were females with an overall mean age of 49.58 ± 10.32 years. Table-II shows the anthropometric, clinical and biochemical determinants of MS with gender differences. The percentage of overweight and obese male subjects as defined by BMI is significantly higher as compared to female population (p< 0.05).No significant difference was found on the basis of central obesity as defined by IDF while with ATP III criterion, female subjects were significantly more centrally obese as compared to male subjects (p < 0.0001). High triglyceride levels, abnormal HDL cholesterol and raised BP were also significantly more prevalent in male diabetic subjects as compared to female subjects(p< 0.05). Consequently the frequency of MS was higher in male study subjects as compared to female subjects. The difference was statistically significant for IDF defined MS(p=0.006). The overall prevalence of MS in study population was 62.2% and 65.5% by IDF and ATP III definitions of MS respectively.

We compared the distribution of genotypes in 296 type 2 diabeticpatients for MS positive and negative population as defined by IDF and ATP III definition. The “A” allele at rs9939609 in the *FTO* gene had a frequency (MAF) of 0.74 in type 2 diabetics. The genotype frequencies did not deviate from the Hardy Weinberg predictions (concerned data is not intended for current analysis). Carriers of ≥ 1 copy of the rs9939609 A allele were significantly more likely to have IDF defined MS (69.6%) than non-carriers (30.4%), corresponding to a carrier odds ratio (OR) of 0.52 (95% confidence interval [CI] (0.29-0.93), with a similar trend for the NCEP ATP III-defined MS. The carriers OR for the hypertension, increased TG, decreased HDL and central obesity was1.6, 0.6, 0.9 and 0.6 respectively for IDF defined MS and 1.6, 0.6, 0.9 and 0.7 for ATP III defined MS respectively. The trend is in favor of carrier of risk allele A for all the determinants of MS which showed statistically significant difference for the allelic distribution among the determinants of MS(Table-III).

**Fig.1:**
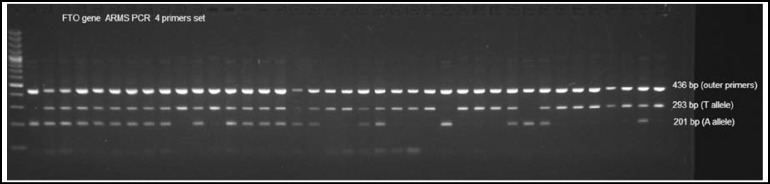
“ARMS PCR” for *FTO* gene.

Fig.1, illustrates the “ARMS PCR” for *FTO* gene showing the separation of A & T allels.

## DISCUSSION

The discovery of *FTO *gene and its researched associations is considered a milestone of Human genome studies.^19^ *FTO* gene has been extensively investigated because of its association with obesity, cardiovascular risk factors and MS.^8^Studies from Asia and specifically from South East Asia reported the association of *FTO* gene with obesity and associated risk factors.^1^^,^^3^ Variant of *FTO* gene at rs9939609 is also found to be associated with patients with type 2 diabetes in Europeans and South Asians.^20^ The association of patients with obesity has been replicated in a Pakistani study.^21^

The first report on association between the *FTO* rs9939609 A allele and MS was published in 2001^8^ and then this associated had been replicated in many studies involving different ethnic groups across the globe.^8^ To the best of our knowledge, this is the first report of an association between the *FTO* rs9939609 A allele and MS from Pakistani diabetic population.

This hospital based study of *FTO* as a candidate gene for MS in a type 2 diabetic sample from a multiethnic population showed significant association with the *FTO* gene (rs9939609 A allele carriers) having an increased risk of MS defined through IDF and ATP III definitions. Similar trends were reported by Al-Attar et al., in a multi-ethnic population study.^8^ A meta-analysis across the four non-Caucasian populations also showed the similar association between MS and the common rs9939609 SNP in *FTO* gene.

This association was linked to higher frequency of subjects with decreased HDL cholesterol and a tendency towards high waist circumference. Similar trends were observed for the ATP III definition of MS.

Further analysis of the sub-factors for MS, indicated that significantly more A allele carriers at *FTO* rs9939609 met the MS criteria for decreased healthy HDL cholesterol than non A allele carriers. In a non-Caucasian population study, significantly more A allele carriers had the MS criteria for decreased HDL cholesterol.^9^Studies conducted in European and Korean population showed that the carriers of A-risk allele at rs9939609 was significantly associated with lower HDL cholesterol.^3^^,^^22^ The significant association of Triglyceride (TG)with the carriers of the A allele in this study is in agreement with other published studies from the region and throughout world.^3^^,^^22^

Significant difference was observed in the analysis of association of A allele with high blood pressure in type 2 diabetic subjects. Blood pressure is considered as the single most important risk factor for Cardiovascular disease especially in diabetes which is already considered as cardiovascular risk equivalent.^23^ This association of A allele of *FTO* gene at rs9939609 with systolic blood pressure has also been replicated in MONICA study.^24^A Korean study also reported that high blood pressure, may be the potential component for the reported association between *FTO*, BMI, and MS.^3^ A Pakistani study reported significant association of rs9939609 variant with blood pressure in adult non-diabetic females and this association remained significant even after adjusting the BMI.^21^

For the association of “A” allele with obesity related traits in diabetic population of this study, the reported values were significant for increased waist circumference. The association of *FTO* gene with central obesity has been extensively investigated and well established. Other reported European and Southeast Asian studies confirm this association^3^^,^^22^. 

The findings from this study indicate that the association of the *FTO* rs9939609 Aallele with the MS was related in part to both the obesity component, but also the HDL and triglycerides component, suggesting that this genetic marker may have a broader relationship with individual components of this complex trait. Although the observations from this study may help to understand the molecular etiology of MS but as this study is hospital based, the association observed needs to be replicated in larger community based studies. Despite insufficient sample size, the observations from this study ascertain the established findings and association between the *FTO* rs9939609 A allele with obesity in previous reported studies.^25^ However, MS is a multifactorial and complex metabolic disorder, and only a few of the reported genes, such as *PPARG* and *APOC3*, have been reproduced in more than one study sample.^26^ Apart from the requirement to reproduce and replicate the SNP at *FTO* gene in other larger study samples, the cellular and molecular mechanics causing this linkage also needs to be researched. The cellular mechanisms may be interrelated to either a direct effect or function of the studied gene i.e. *FTO* gene^27^ or they may be due to the linkage disequilibrium of the targeted gene variant with another pathological change in a gene leading to mutation in the *FTO* locus.

Insufficient sample size and involving patients from a single tertiary care unit without a control population are the limitations of this study which may affect the true implications of this study to the general public. However, with scarce reported data on genetic association from Pakistan, this study may still be worthwhile to conduct well planned future studies accounting all these shortfalls. It is also worthwhile to note that treatment affecting the control of diabetes can affect the phenotype of MS. Targeting the untreated, newly diagnosed diabetics can minimize this effect.

**Table-I:** Metabolic syndrome classification according to IDF and ATP III criteria

**IDF clinical criteria** *(Central Obesity and any two of other four criteria constitute a diagnosis of Metabolic Syndrome)*	**ATP III: clinical criteria** *(Any three of other five criteria constitute a diagnosis of Metabolic Syndrome)*
**Central Obesity** Waist circumference ≥ 90cm for South Asian men Waist circumference ≥ 80cm for South Asian women	**Central Obesity** Waist circumference ≥ 102 cm for South Asian men Waist circumference ≥ 88 cm for South Asian women
**Serum triglyceride** ≥ 150 mg/dl (1.7 mmol/L), or specific treatment for this lipid abnormality	**Serum triglyceride** ≥ 150 mg/dl (1.7 mmol/L), or specific treatment for this lipid abnormality
**HDL cholesterol** < 40 mg/dl (1.0 mmol/L) in males, < 50 mg/dL (1.3 mmol/L) in females or specific treatment for this lipid abnormality	**HDL cholesterol** < 40 mg/dl (1.0 mmol/L) in males, < 50 mg/dL (1.3 mmol/L) in females or specific treatment for this lipid abnormality
**Hypertension** Blood Pressure ≥ 130/85 mm Hg, or treatment of previously diagnosed hypertension	**Hypertension** Blood Pressure ≥ 130/85 mm Hg, or treatment of previously diagnosed hypertension
**Impaired fasting glucose** ≥ 100 mg/dL (5.6 mmol/L), or previously diagnosed type 2 diabetes	**Impaired fasting glucose** ≥ 110 mg/dl (5.6 mmol/L), or previously diagnosed type 2 diabetes

**Table-II:** Clinical and Biochemical data of the study participants

**Variables**	**Overall** **n=296**	**Male** **n=179 (60.47%)**	**Female** **n=117 (39.52%)**	**p-value**
Age	49.58 ± 10.32	50.05 ± 10.59	48.87 ± 10.04	0.341
Body mass index (%)				
Obese	77.8	57	43	0.0027
Over weight	13	69.4	30.6	0.0010
Normal	9.8	72.5	27.5	0.0006
Waist circumference (%) (MS IDF)				
Central obese	79	52.7	47.3	0.2526
Normal	21	89.4	10.6	<0.0001
Waist circumference (%)(MS ATP III)				
Central obese	50	34.5	65.5	<0.0001
Normal	50	86.3	13.7	<0.0001
MS Triglycerides (%)				
Increased	77.7	68.2%	31.8	<0.0001
Normal	22.3	44.4%	55.6	0.2482
MS High density lipoprotein (%)				
Reduced	90	64	36	<0.0001
Normal	10	50	50	1.0000
MS Blood pressure (%)				
Increased	65.5	59.3	40.7	0.0003
Normal	34.5	62.7	37.3	0.0003
Metabolic Syndrome (%)				
IDF	184/296 62.2	100/184 54.4	84/184 45.6	0.0953
ATP III	194/296 65.5	111/194 57.3	83/194 42.7	0.0045

Data presented Mean ± SD and%

**Table-III:** Metabolic syndrome and metabolic syndrome component prevalence in participant when classified in according to their genotype of the *FTO* rs9939609T>A polymorphism byusing dominant model

	**TT** **n=77** **(26%)**	**AA &AT** **n=219** **(74%)**	* **OR95%CI**	** **p-value**
**IDF**				
IDF MS(%)	30.4	69.6	0.52(0.29-0.93)	<0.0001
Male (%)	36.0	64.0	0.55(0.29-1.06)	<0.0001
Female (%)	23.8	76.2		<0.0001
Central obesity (%)	27.7	72.3	0.69(0.34-1.40)	<0.0001
Hypertension (%)	22.7	77.3	1.63(0.95-2.78)	<0.0001
Fasting blood sugar (%)	32.0	68.0	1.65(0.56-4.85)	<0.0001
High density lipoprotein (%)	26.7	73.3	0.91(0.34-2.41)	<0.0001
Triglycerides (TG)(%)	28.6	71.4	0.64(0.30-1.33)	<0.0001
ATP III				
ATP III MS (%)	27.3	72.7	0.81(0.46-1.42)	<0.0001
Male (%)	29.7	70.3	0.75(0.39-1.43)	<0.0001
Female (%)	24.1	75.9		<0.0001
Central obesity(%)	28.8	71.2	0.77(0.45-1.31)	<0.0001
Hypertension (%)	22.7	77.3	1.63(0.95-2.78)	<0.0001
Fasting blood sugar (%)	31.9	68.1	1.55(0.56-4.25)	<0.0001
High density lipoprotein (%)	26.7	73.3	0.91(0.34-2.41)	<0.0001
Triglycerides (TG)(%)	28.6	71.4	0.64(0.30-1.33)	<0.0001

* *Odds ratio by Chi-square.*

** *p-value calculated by Z-test.*

Finally to conclude the association of “A” allele at rs9939609 in *FTO* gene is found to be significant with MS and its determinants. To ascertain this relationship, the findings from this study need to be replicated in a larger, case control and community based studies.
